# Editorial Comment to Survival beyond cabazitaxel for metastatic castration‐resistant prostate cancer

**DOI:** 10.1111/iju.15458

**Published:** 2024-04-04

**Authors:** Yoshiyuki Yamamoto, Norio Nonomura

**Affiliations:** ^1^ Department of Urology Osaka University Graduate School of Medicine Osaka 565‐0871 Japan

The authors reported the survival beyond cabazitaxel for metastatic castration‐resistant prostate cancer (mCRPC).^1^ In this report, real world data showed that the prognosis was better if any treatment for mCRPC was administered after cabazitaxel. Especially, patients who received first‐line androgen receptor signaling inhibitors (ARSI), radium‐223, olaparib or docetaxel rechallenge after cabazitaxel presented longer OS than those who did not receive subsequent therapy or other treatments with alternative ARSI or estrogen.^1^ Similar reports have been relatively rare, and this report is clinically meaningful.

Recently, the landscape for mCPRC therapy has rapidly evolved with the approval of several agents including ARSIs such as abiraterone, enzalutamide, and darolutamide, taxanes such as docetaxel and cabazitaxel, and radium‐223.^2^ However, no randomized controlled trial comparing these treatments has existed, thus the optimal treatment sequence remains unknown. The European Association of Urology and National Comprehensive Cancer Network guidelines do not clearly state an optimal sequence for mCRPC treatments.^2^

The Card trial showed that mCRPC patients with cabazitaxel after first‐line ARSI and docetaxel showed better progression‐free survival and overall survival (OS) than those with the second‐line ARSI.^3^ On the other hand, few reports exist, especially regarding post‐cabazitaxel therapy. An Australian study showed longer OS in patients who received subsequent treatment beyond cabazitaxel than in those without further therapy after cabazitaxel.^4^ This difference in OS can be explained by the survival benefit of effective treatment even after cabazitaxel and has an important clinical meaning in the management of patients with mCRPC. This finding is supported by recent evidence from the VISION trial, which revealed survival benefit of 177Lu‐PSMA‐617 after taxanes including cabazitaxel.^5^ Therefore, further research is required to know the role of each agent after cabazitaxel to prolong OS.

This study was retrospective, and 36 cases were not sufficient. The variety of post‐cabazitaxel treatments made interpretation regarding OS improvements difficult. It is necessary to consider the possibility that patients who could undergo any post‐cabazitaxel therapies had relatively good general health and cancer status, which might have led to a favorable prognosis. However, it is generally challenging to obtain sufficient background factors in these late treatment line of post‐cabazitaxel. In the future, further evidence building is expected through the accumulation of cases at multiple centers in the absence of established post‐cabazitaxel therapy.

## CONFLICT OF INTEREST STATEMENT

None declared.
